# Incidental dural tears during pediatric posterior spinal fusions

**DOI:** 10.1007/s43390-024-00873-4

**Published:** 2024-05-23

**Authors:** Paal K. Nilssen, Edward Compton, Stephen Stephan, Lindsay M. Andras, Jason K. Chu, David L. Skaggs, Kenneth D. Illingworth

**Affiliations:** 1 Cedars-Sinai Medical Center, Department of Orthopedics, 444 S San Vicente Blvd #901, Los Angeles, CA 90048 USA; 2 Children’s Orthopaedic Center, Children’s Hospital Los Angeles, Los Angeles, CA USA; 3 Department of Orthopedics, Scripps Clinic, La Jolla, San Diego, CA USA; 4 Division of Neurosurgery, Department of Surgery, Children’s Hospital of Los Angeles, Los Angeles, CA USA

**Keywords:** Pediatric, Dura tears, Outcomes, Complications, Spine

## Abstract

**Purpose:**

To characterize the frequency of incidental dural tears in pediatric spine surgery, their treatment, complications, and results of long-term follow-up.

**Methods:**

A retrospective review of all pediatric patients who underwent a posterior spinal fusion (PSF) between 2004–2019 at a tertiary children’s hospital was conducted. Electronic medical records were reviewed for patient demographics, intra-operative data, presence of an incidental dural tear, repair method, and patient outcomes.

**Results:**

3043 PSFs were reviewed, with 99 dural tears identified in 94 patients (3.3% overall incidence). Mean follow-up was 35.7 months (range 0.1–142.5). When the cause of the dural tear was specified, 69% occurred during exposure, 5% during pedicle screw placement, 4% during osteotomy, 2% during removal of implants, and 2% during intra-thecal injection of morphine. The rate of dural tears during primary PSF was significantly lower than during revision PSF procedures (2.6% vs. 6.2%, p < 0.05). 86.9% of dural tears were repaired and/or sealed intraoperatively, while 13.1% had spontaneous resolution. Postoperative headaches developed in 13.1% of patients and resolved at a mean of 7.6 days. There was no difference in the incidence of headaches in patients that were ordered bedrest vs. no bedrest (p > 0.99). Postoperative infections occurred in 9.5% of patients and 24.1% patients were identified to have undergone a revision surgery.

**Conclusions:**

Incidence of intra-operative dural tears in pediatric spine surgery is 3.3%. Although complications associated with the dural tear occur, most resolve over time and there were no long-term sequelae in patients with 2 years of follow up.

**Level of evidence:**

Level IV.

## Introduction

Incidental dural tears are a known complication of spinal surgery. In the pediatric population, the rate has been reported between 0.35 to 3.5% for primary surgeries [1, 2]. However, the incidence significantly increases during revision surgeries, which has been reported to be as high as 18.5% [1–4]. This increase in incidence is thought to be due to adhesions in the epidural space, coupled with scarring and fibrosis of the dura itself [1–3, 5]. When dural tears are left untreated, cerebrospinal fluid (CSF) leaks can lead to inadequate cushioning of the brain and increased tension on the brain’s anchoring structures [6]. When standing, this can lead to neurological sequelae, such as postural headaches, nausea, vomiting, pain or tightness in the neck and back, dizziness, diplopia, photophobia, tinnitus, and blurry vision [6–8]. In addition to neurological side effects, sequelae such as pseudomeningoceles, CSF fistulas, neurological deficits, durocutaneous fistulas, meningitis, bladder/bowel dysfunction, and cauda equina syndrome have also been documented [2, 9–14].

When incidental dural tears occur, several studies advocate for immediate repair if identified intra-operatively and for return to the operating room for surgical repair if identified post-operatively [9, 10, 12, 13]. In the adult population, immediate suture repair has demonstrated resolution of neurological symptoms and equivalent outcomes compared to patients without tears [9, 12, 13]. In addition to direct repair, some studies also advocate for mandatory bedrest while others advocate for natural postoperative ambulatory progression if asymptomatic form the CSF leak [9, 10, 12–14]. In pediatric spinal deformity patients, the etiology and management of dural tears is not well documented with the largest prior study only including 6 patients [2]. The purpose of this study is to characterize the cause, treatment modality, and outcomes of incidental dural tears in pediatric patients undergoing posterior spinal fusion (PSF).

## Methods

A retrospective review of pediatric patients with a spine deformity etiology who underwent PSF between 2004–2019 at a tertiary children’s hospital was conducted. All patients underwent surgery by a board-certified orthopedic surgeon or neurosurgeon specializing in pediatric spine. Surgical assistants ranged from PGY-2 orthopedic residents to spine fellows. Navigation was not used in any surgery.

Demographic information, preoperative diagnosis, prior spine surgeries, method of dural tear repair, post-operative management, and complication data were extracted from medical records. A dural tear within the surgical field in patients with prior spine surgery was defined as any past procedure exposing the spine canal. For scoliosis patients, durotomy location was categorized concerning spine curvature: apex (the vertebral body with the largest lateral deviation from the midline), periapical region (1–2 vertebral bodies inferior or superior to the apex), and upper/lower junctional regions (any vertebral bodies extending beyond the periapical regions).

Post-operative management data included drain placement and mandatory bedrest. Notably, the decision to place postoperative drains was at the discretion of the treating physician, considering factors like blood loss, dural tear size, repair quality, and adjacent tissue condition. Symptoms of CSF leaks, including headaches, nausea, vomiting, dizziness, and lightheadedness, were recorded. Complications, such as neurological deficits, pseudomeningoceles, persistent wound drainage, post-operative infections (surgical site infections, meningitis, etc.), and recurrence of CSF leak were documented. Patients with over 2 years follow-up were analyzed separately for long-term outcomes, including revision surgeries, late infections, and wound problems.

Statistical analyses were performed using STATA/1C 14.0 (Stata Statistical Software: Release 14; StataCorp LP, 2015, College Station, TX). Statistical significance was determined at a P value of < 0.05. Descriptive statistics, such Fisher’s exact test and student t-tests were used to compare categorical and continuous variables, respectively. Institutional review board approval was granted for this study.

## Results

### Patient demographics and incidence

At our hospital, 3,045 PSFs were reviewed, with 99 dural tears identified in 94 patients (3.3% overall incidence). Female patients comprised 57.6% (n = 57) of patients and the average age was 13.2 ± 4.1 years (range: 3.4–22.7). The average height and weight at the time of surgery was 139.2 ± 25.4 cm and 42.1 ± 22.9 kg, respectively. The incidence of dural tears by operative indication, procedure type, and location are presented in Table 1. When the cause of the dural tear was specified, 83.0% (n = 73/88) occurred during exposure, 5.7% (n = 5/88) during pedicle screw placement, 4.5% (n = 4/88) during osteotomy, 2.3% (n = 2/88) during removal of implants, and 2.3% (n = 2/88) during intra-thecal injection of morphine. In the cervical spine, 9 dural tears were identified (all < 3 mm in size): 4 occurred secondary to scar tissue adhering to the dura on exposure, 3 during exposure (unspecified), and 2 unknown. Five were repaired with sutures, 3 with adjuvant treatments, and one spontaneously resolved.

**Table 1:** Rate of dural tears by operative indication, procedure type, and location

	N (%)	P-value
**Operative Indication**		
Type of Scoliosis		< 0.05
Neuromuscular	48/736 (6.5)	
Congenital	17/415 (4.1)	
Syndromic	12/331 (3.6)	
Idiopathic	9/1344 (0.7)	
Spondylolisthesis	5/106 (4.7)	
Trauma	8/111 (7.2)	
**Procedure Type**		< 0.05
Primary	63/2,463 (2.6)	
Revision	36/582 (6.2)	
**Location of Dural Tear**		< 0.05
Cervical	9 (9.1)	
Thoracic	30 (30.3)	
Lumbar	38 (38.3)	
Sacral	12 (12.1)	
Not Specified*	10 (10.1)	

^*^All ten of these patients underwent PSFs with upper instrumented vertebrae in the thoracic spine and lower instrumented vertebrae in the lumbar or sacrum

The rate of dural tears during primary PSF were significantly lower than during revision PSF (2.6%, 63/2463 vs. 6.2%, 36/581; p < 0.05). During revision PSF, 69% (n = 25/36) of dural tears occurred during exposure, 8.3% (3/36) during removal of hardware, 5.6% (2/36) during osteotomy, 2.7% (1/36) during pedicle screw placement, 2.7% (1/36) during a intrathecal duramorph injection, and 11.1% (4/36) were unknown. Of the patients who had a previous spine surgery, 53.5% (23/43) of dural tears occurred within the previous surgical field, 34.9% (15/43) were outside the previous surgical field, and 11.6% (5/43) could not be determined from the operative note. Patients with a history of a neurosurgical procedure in which the spinal column was exposed, such as a Chiari decompression, laminectomy, or spinal cord detethering, had higher rates of dural tears (3.2% vs. 9.1%, p < 0.05) during the subsequent surgery due to the dura becoming adherent to scar tissue.

### Intraoperative repair

Out of the 99 dural tears, 86 (86.9%) were repaired and/or sealed intra-operatively. Direct repair with suturing was accomplished in 71 (71.7%) cases with some requiring adjuvant treatments, such as fibrin sealant (n = 15), collagen scaffold (n = 13), polyethylene glycol sealant (n = 7), and muscle graft (n = 1). In the remaining 15 (15.2%) patients treated at the time of surgery, direct repair was not achieved. Among those patients, the tear was addressed as follows: collagen scaffold alone (n = 5), fibrin sealant alone (n = 2), collagen scaffold with fibrin sealant (n = 2), collagen scaffold with fibrin sealant and muscle graft (n = 1), polyethylene glycol sealant (n = 1), gelfoam (n = 2), bonewax (n = 1), and thrombin (n = 1). Lastly, 13 (13.1%) cases had spontaneous resolution of the CSF leak by the end of the procedure and no repair was attempted. In all cases, there were no intra-operative neuromonitoring changes associated with the dural tear.

### Perioperative management

Drains were placed in 65 (65.7%) patients, of whom 26 had a drain placed superficial to the fascial layer, 25 deep to the fascial layer, and 10 both deep and superficial to the fascial layer. In 4 drains, the depth was not specified. Drains remained in place until the output was < 30 cc/24 h. If there were concerns for a persistent CSF leak, drains were placed to gravity rather than suction. Mandatory bedrest was ordered for 43 (43.4%) patients after their surgery for an average of 2.1 ± 1.7 days (range: 1–12 days). Of those patients, 7 experienced symptoms associated with a CSF leak and were subjected to slightly longer durations of bedrest (average: 4.7 ± 2.8 days).

### Peri-operative sequelae

Postoperative headaches developed in 13 (13.1%) patients, which resolved at a mean of 7.6 ± 4.6 days (range: 2–17). The incidence of headaches did not differ whether the patient underwent suture repair (13.3%, 4/30 vs. 12.2%, 9/74; p = 0.84), drain placement (12,1%, 8/66 vs. 15.8%, 6/38; p = 0.77), or bedrest (12.5%, 5/40 vs. 14.1%, 9/64; p = 0.81). Postoperative nausea developed in 31 (31.3%) patients, which resolved at a mean of 3.2 ± 2.2 days (range: 1–8) after the procedure. Similarly, the incidence of nausea did not differ whether the patient underwent suture repair (32.4%, 23/71 vs. 23.3% 7/30; p = 0.42), drain placement (35.4%, 23/65 vs. 21.1%, 8/38; p = 0.18), or bedrest (29.7%, 16/64 vs. 30.0%, 12/40; p = 0.67).

Four patients that underwent intra-operative repair of their dural tear had secondary procedures to address persistent CSF leaks: 2 patients were treated with placement of drains, 1 patient had revision dural repair, and 1 patient had surgical exploration with no CSF leak identified and no further leakage. The 15 patients with spontaneous resolution during surgery had a similar rate of postoperative headaches (13.3%; 2/15) as those who had a repair (13.5%; 12/89) (p > 0.999), none of which (0/15) required any further intervention. Surgical site infections occurred in 10 (9.6%) patients. Of those, 7 received antibiotics and underwent repeat irrigation and debridement until resolution of their infection. The other 3 (all with deep infections) required antibiotics, irrigation and debridement, and revision of instrumentation. Two patients developed post-operative pseudomeningoceles, which were repaired surgically. The average length of stay for patients who had dural tears was 10.2 ± 12.6 days.

### Long-term sequelae

The average length of follow-up for all 3,043 procedures was 35.7 ± 34.9 months. Patients who had a dural tear had similar lengths of follow-up (33.1 ± 30.2 months). Among patients with a dural tear, 52 (52.5%) had follow-up durations longer than 2 years (mean: 54.2 ± 28.2 months, range: 24.4–142.5). Thirteen (24.1%) patients were identified to have undergone a revision surgery at an average of 12.5 ± 20.0 months (range: 0.6–72.1) for the following reasons: implant failure (n = 5), pseudoarthrosis (n = 2), recurrence/progression of deformity (n = 2), postoperative infection in the perioperative period (n = 2), distal junctional kyphosis (n = 1), and nonunion (n = 1). Lastly, 1 patient developed infected hardware 5 years after the initial procedure, which was treated with irrigation and debridement, with no further sequelae.

## Discussion

Incidental dural tears are known complications of pediatric spine surgery (reported rates: 0.35–3.5%) [1–4]. Numerous reports are present in the adult spine literature, but few studies have detailed incidental dural tears in the pediatric spine population. This series of 3,043 pediatric PSFs, represents the largest study of incidental dural tears in the pediatric literature. In this study, we found that incidental durotomies had an incidence rate of 3.3%, consistent with previous studies, and occurred significantly more often during revision procedures. Headaches and nausea were routinely reported by patients after sustaining a tear, regardless of whether a repair was attempted, length of bedrest, or drain placement. Symptoms often resolved by post-operative day 4 and 8, respectively, without recurrence. Lastly, 9.6% of patients suffered post-operative surgical site infections, which is similar to previously reported rates in pediatric spine deformity correction [15–17]. We attribute this rate to the high proportion of neuromuscular patients in our cohort, which has been shown to have increased rates of infection compared to other etiologies of scoliosis [17].

When incidental dural tears occur, a number of methods of repair exist, including direct suture repair alone, direct suture repair with an adjuvant sealant, or repair with a sealant alone. In the present study, the method of repair was at the discretion of the surgeon, who decided based on anatomical location and size of the tear. The gold standard remains direct suture repair, with Eismont et al*.* advocating for diligent and complete closure of dural tears when recognized at the time of surgery, with testing of the integrity of the seal via the Trendelenberg test and Valsalva maneuver [10]. Neglecting to repair a dural tear that does not spontaneously resolve would likely predispose patients to fistula formation, with the possibility of meningitis, pseudomeningoceles, back pain, headaches, and/or nerve root entrapments. However, in some cases, direct suture repair is not possible when the dura is not accessible, in which case the use of an adjuvant may be necessary. In this study, we found that patients who underwent direct suture repair had similar rates of post-operative headaches and nausea as patients that did not undergo direct suture repair. Furthermore, we found patients that suffered small dural tears that were deemed to resolve spontaneously by the surgeon did not lead to adverse patient outcomes. Therefore, when direct suture repair is not possible due to anatomical location, a sealant, such as collagen matrix, fibrin, or polyethylene glycol appear to be viable alternatives.

Recently, the routine use of post-operative drains in patients with dural tears has been called into question by some authors due to fear of the formation of durocutaneous fistulas [10]. However, Wang et al. found that subfascial drains did not lead to durocutaneous fistulas in any of their 88 patients with dural tears[2]. Likewise, Cammisa et al. found that post-operative drains did not lead to any adverse patient outcomes [9]. In the current study, we found no patients experienced negative outcomes secondary to drain placement. Additionally, patients with drains were not more likely to experience post-operative symptoms such as headaches or nausea. Therefore, our findings suggest if a surgeon is not confident in the quality of the dural tear repair and surrounding tissues, a drain may be placed without additional post-operative morbidity.

The length of bedrest following a dural tear has been debated, with some authors advocating for mandatory bedrest, as the lower hydrostatic pressure of the CSF against the dura when lying flat may facilitate accelerated healing [9, 10, 12, 13]. On the other hand, bedrest may unnecessarily lead to longer length of stays and added medical costs [14]. In our study, we found that only 43.4% (43/99) of patients were placed on mandatory bedrest after surgery. Of these, 36 were prescribed mandatory bedrest beginning post-operative day zero, while 7 were prescribed mandatory bed rest after developing symptoms. No difference was observed in the rate of nausea and headaches between patients placed on mandatory bedrest beginning post-operative day zero and patients not placed on mandatory bedrest immediately after surgery. Similarly, Hodges et al. reviewed 20 patients with incidental dural tears that were allowed to ambulate according to their standard postoperative protocol following spine procedures and found that 15 were asymptomatic, 2 had headaches, 2 had nausea, and 1 experienced tinnitus [15]. Therefore, mandatory bedrest following surgery may not be necessary. The authors advocated that most patients should be allowed to ambulate according to the natural ambulatory progression of their procedure, with bedrest being reserved for those that develop symptoms.

Lastly, incidental dural tears in the pediatric population resulted in a 24.1% revision rate. Although we do not have a control group for comparison, one can certainly hypothesize that this would be much lower than 24.1%. In the pediatric population, long-term outcomes following dural tears are not well documented. In the adult population, Wang et al. found that long-term outcomes of patients with incidental dural tears were similar to those without dural tears [13]. Of 88 procedures that resulted in a dural tear, 76 (86.4%) had a good or excellent result, 9 (10.2%) had a satisfactory result, and 3 (3.4%) had a poor result. The authors concluded that a dural tear can almost always be repaired primarily, with a good or excellent outcome without additional complications. Similar to Wang et al. we also advocate for primary repair if the dural tear was recognized intra-operatively.

There are several limitations to this study. First, this is a retrospective chart review of patients from a single center’s experience. Consequently, we were limited by the amount of detail provided in the operative reports and medical records and were unable to expound on specific technical information about the injury mechanism of the dural tear. Additionally, retrospective reviews may underreport rates of complications such as headaches and nausea, as they rely on accurate documentation within the patient’s chart. Additionally, almost half of the patients within our study cohort had neuromuscular disease, which may have affected the patients’ ability to communicate their symptoms. Severe accompanying underlying neurocognitive defects may have led to underreporting of symptoms such as headache and nausea, as these findings are subjective and not elicited on physical exam. Documentation of mandatory bedrest may have been underreported in patient charts but communicated to the nursing and physical therapy staff. Likewise, as many patients had neuromuscular disease, they may not have been ambulatory at baseline, facilitating bedrest even though it was not documented in the patient charts. In the future, larger, multicenter studies ought to be done to mitigate local practice pattern biases.

Overall, the rate of incidental dural tears during pediatric spine surgery is 3.3%. The majority of dural tears occur during exposure of the spine, with few instances occurring during instrumentation. When addressed intra-operatively with direct suture repair, sealant, or are deemed to self-resolve, pediatric patients appear to tolerate dural tears well. Pediatric patients rarely develop persistent CSF leaks or pseudomeningoceles after incidental dural tears. Mandatory bedrest does not appear to be necessary, as patients with normal post-operative ambulatory progression had satisfactory clinical outcomes.

## Data Availability

Data is available from the corresponding author for a reasonable request.
